# Nesidioblastosis in Pregnancy: Navigating the Diagnostic and Therapeutic Challenges of a Rare Condition

**DOI:** 10.7759/cureus.71985

**Published:** 2024-10-21

**Authors:** Basel Darawsha, Ayat Agbaria, Polina Stein, Safi Khuri

**Affiliations:** 1 General Surgery, Rambam Medical Center, Haifa, ISR; 2 Pediatrics, Ruth Rappaport Children's Hospital, Rambam Medical Center, Haifa, ISR; 3 Pathology, Rambam Medical Center, Haifa, ISR; 4 Pancreato-Biliary Surgery Service, Hepato-Pancreato-Biliary and Surgical Oncology Unit, Rambam Medical Center, Haifa, ISR

**Keywords:** adult nesidioblastosis, gradual pancreatectomy, hyperinsulinism, insulinoma, nesidioblastosis in pregnancy, niphs, total pancreatectomy

## Abstract

Nesidioblastosis, a non-neoplastic proliferation of the pancreatic islet cells of Langerhans, is a rare cause of endogenous hyperinsulinemic hypoglycemia. Although initially thought of as a congenital disease affecting pediatric patients, it is well known nowadays to affect adults as well. In addition, it is increasingly documented as a rare sequela of bariatric surgeries. Management options include medical and surgical therapies, with little known about the beneficial effects of both. Nesidioblastosis affecting pregnant patients is even rarer, with scarce literature known about the optimal treatment.

Herein, we present a 22-year-old woman in the 20th week of gestation who experienced symptomatic episodes of severe life-threatening hypoglycemia. The management of this case required a multidisciplinary team approach to navigate the complexities of diagnosis and treatment. The complexity of this case was further heightened by the differential diagnosis, including conditions like insulinoma. The scarcity of literature on nesidioblastosis in pregnancy further complicated the case, underscoring the need for more research and case studies to guide clinical practice.

## Introduction

Nesidioblastosis, first described in 1938 by George F. Laidlaw, is an extremely rare medical disease that leads to hyperinsulinemic hypoglycemia [1]. In adults, the most frequent cause of pancreatic hyperinsulinemic hypoglycemia is insulinoma [2], but nesidioblastosis remains in the differential diagnosis when no detectable mass is visible on various imaging techniques.

Nesidioblastosis is a non-neoplastic proliferation of the islets of Langerhans from the pancreatic ductal epithelium. The neoformation of these islets of Langerhans was initially described in the pediatric age group of patients. This entity was thought to be a congenital disorder occurring in neonates and infants. Over the years, it has been expanded to include an adult version of this condition. It accounts for approximately 0.5% to 5% of adult hyperinsulinemic hypoglycemia etiologies [3]. Notably, after the widespread adoption of bariatric surgery in the past two decades, nesidioblastosis has been increasingly documented as a rare sequela of gastric bypass [4].

In this case report, we present a 22-year-old woman in the 20th week of gestation who experienced symptomatic episodes of severe life-threatening hypoglycemia. The management of this case required a multidisciplinary team approach to navigate the complexities of diagnosis and treatment. The complexity of this case was further heightened by the differential diagnosis, including conditions like insulinoma. The scarcity of literature on nesidioblastosis in pregnancy further complicated the case, underscoring the need for more research and case studies to guide clinical practice. This report highlights the diagnostic challenges, the required multidisciplinary efforts, and the limited treatment options for managing nesidioblastosis in pregnant women.

## Case presentation

A 22-year-old primigravida at 20 weeks gestation, conceived through IVF, presented to the emergency department with severe lethargy. Her medical history was significant for multiple sclerosis and status post (S/P) sleeve gastrectomy, later converted to a mini gastric bypass due to weight regain. The patient reported experiencing lethargy, trembling, and presyncope sensation postprandially. Initial point-of-care glucose testing revealed a 46 mg/dL blood glucose level. Following intravenous dextrose administration, the patient experienced a resolution of her symptoms.

Upon presentation to the emergency room (ER) department, the patient's vital signs showed a blood pressure of 101/60 mmHg, a heart rate of 80 bpm with normal oxygen saturation, and no fever. The initial glucose measurement was 60 mg/dL. During her stay in the ER, recurrent hypoglycemic episodes were observed, with glucose levels dropping as low as 36 mg/dL, accompanied by neurological symptoms.

The patient was admitted to the internal medicine ward with a presumptive diagnosis of dumping syndrome, given her history of bariatric surgery. Initial laboratory studies were unremarkable. However, during hospitalization, the patient experienced persistent hypoglycemic episodes unrelated to food intake, necessitating further diagnostic evaluation. A critical sample obtained during a hypoglycemic event revealed elevated levels of C-peptide (774 pmol/L; normal range during hypoglycemia: <100 pmol/L) and insulin (21 μIU/mL; normal fasting range: 2-6 μIU/mL) (Table 1). These findings were inconsistent with the initial diagnosis and suggested inappropriate endogenous insulin secretion, prompting a reevaluation of the patient's condition.

**Table 1:** Critical sample during hypoglycemia * normal range during hypoglycemia, ** normal fasting range

Critical sample	Measured value	Reference range
C-peptide	774 pmol/L	<100 pmol/L*
Insulin	21 μIU/mL	2-6 μIU/mL**

**Figure 1:**
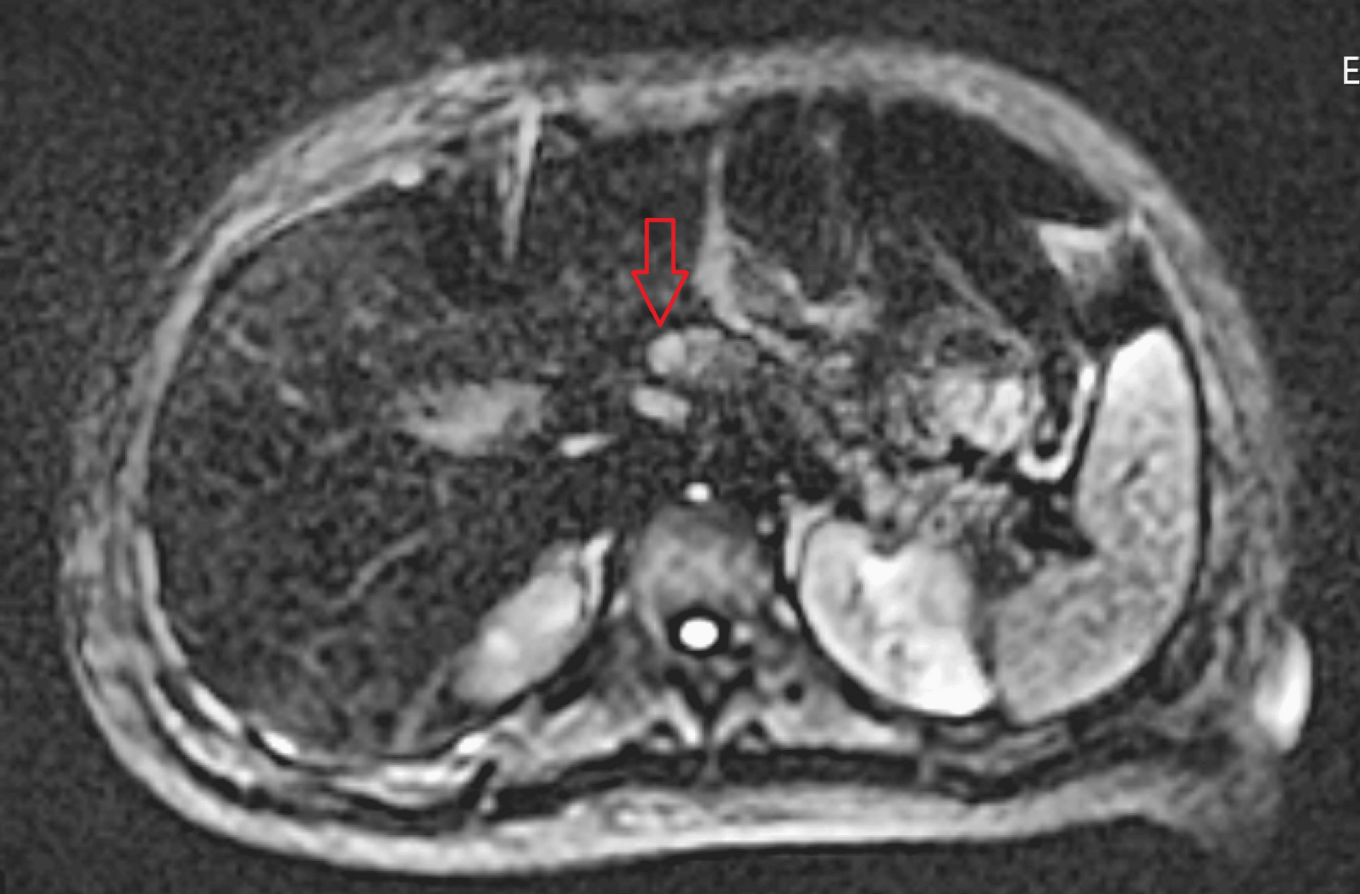
MRCP showing a focus of restricted diffusion at the superior aspect of the pancreatic neck MRCP: magnetic resonance cholangiopancreatography Arrow: suspected insulinoma

The serum C-peptide and insulin levels raised the suspicion for pancreatic insulinoma as a cause for recurrent hypoglycemia. Thus, magnetic resonance cholangiopancreatography (MRCP) was conducted to investigate the possibility of an insulinoma. The MRI revealed a focus of restricted diffusion at the superior aspect of the pancreatic neck, suggesting a pancreatic lesion potentially consistent with an insulinoma (Figure 1). The fact that the patient had S/P mini-gastric bypass and endoscopic ultrasound was unavailable and non-informative.

Given the complex nature of the case, a comprehensive multidisciplinary team was convened to discuss treatment approaches. This team included internal medicine specialists, endocrinologists, pancreatic surgeons, gynecologists, neonatologists, and perinatologists, ensuring that all aspects of the patient's high-risk pregnancy were thoroughly addressed. The team considered medical management using diazoxide, with the potential addition of somatostatin analogs if necessary. However, it was noted that somatostatin analogs and diazoxide may be teratogenic for the fetus, an aspect that will be elaborated upon later in this article. Surgical intervention, specifically a laparoscopic enucleation of the focal lesion identified on MRI, was also proposed as a treatment option. After presenting the potential teratogenic side effects of the medical approach to the patient, she firmly rejected this option. The surgical approach was then thoroughly discussed, including the potential risks and side effects of anesthesia and surgery on both the patient and the fetus. Despite these risks, the patient ultimately decided to proceed with the surgical approach.

The patient underwent exploratory laparoscopy two weeks post-admission. Intraoperatively, examination revealed a grossly normal pancreas, with no lesions identified macroscopically or via intraoperative ultrasound. Palpation through a wound retractor device was also performed, showing no palpable lesions. An intraoperative venous sampling of the splenic and the portal vein for C-peptide and insulin levels was non-conclusive for localizing secretory pancreatic mass. These negative findings significantly decreased the likelihood of insulinoma. Intraoperative consultation with the attending endocrinologist and the surgical team concluded that progressing to a more aggressive form of pancreatectomy might have consequences that outweigh the potential benefits. Consequently, the procedure remained purely exploratory, with no tissue resection performed.

During the postoperative period, rigorous glucose monitoring revealed recurrent hypoglycemic episodes. The patient was managed with a continuous infusion of 10% dextrose at 300 mL/hour via a peripherally inserted central catheter (PICC) line, with 50% dextrose available as needed. Despite dietary modifications and a dextrose treatment regimen, the patient continued to experience hypoglycemic episodes, necessitating additional boluses of intravenous dextrose. Following these persistent findings, a diagnosis of noninsulinoma pancreatogenous hypoglycemia syndrome (NIPHS) was made.

Recurrent hypoglycemic episodes persisted, with the patient's daily dextrose requirement remaining as high as 1460 grams. The refractory hypoglycemia necessitated continuous glucose infusion, posing an ongoing risk to maternal health and fetal development. Given the substantial dextrose requirements and persistent hypoglycemia despite conservative management, the multidisciplinary team was reconvened, and an ethics committee was consulted to discuss further management options. Due to the patient's firm refusal of pharmacological treatment, the surgical team proposed a plan of gradual pancreatectomy. The challenging nature of this procedure, particularly in a pregnant patient, was explicitly presented to the patient. Despite these risks, given the limited alternatives available, it was the most viable option.

**Figure 2:**
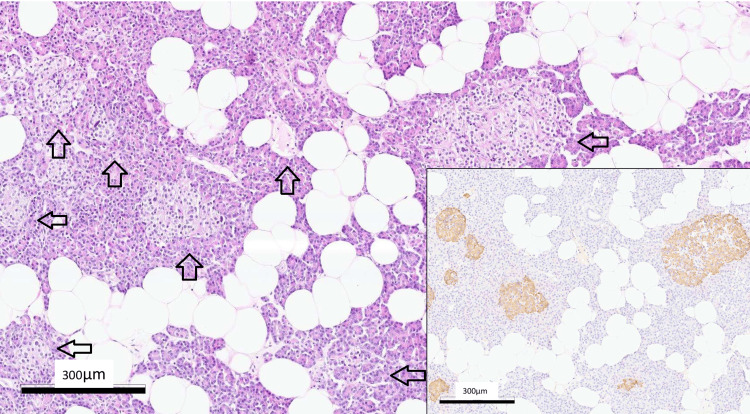
Pancreatic tissue showing the marked hyperplasia of islets, highlighted by insulin immunostaining (inlet). Hematoxylin and eosin staining (x100) and immunoperoxidase (x100). Scale bar =300 μm Arrow: Langerhans islands. Note the increased size and number of islets compared to the normal pancreatic tissue in Figure 3.

At 24 weeks of gestation, the patient underwent a spleen-preserving subtotal pancreatectomy, which was performed without any perioperative complications for the patient or her fetus. Postoperatively, fetal well-being was assessed using cardiotocography and ultrasound examinations, demonstrating reassuring fetal heart rate patterns and normal fetal movement and showing no adverse effects of the surgery or anesthesia on the fetus. The final histopathological report from the resected pancreatic tissue was consistent with the marked hyperplasia of Langerhans islets (Figure 2).

**Figure 3:**
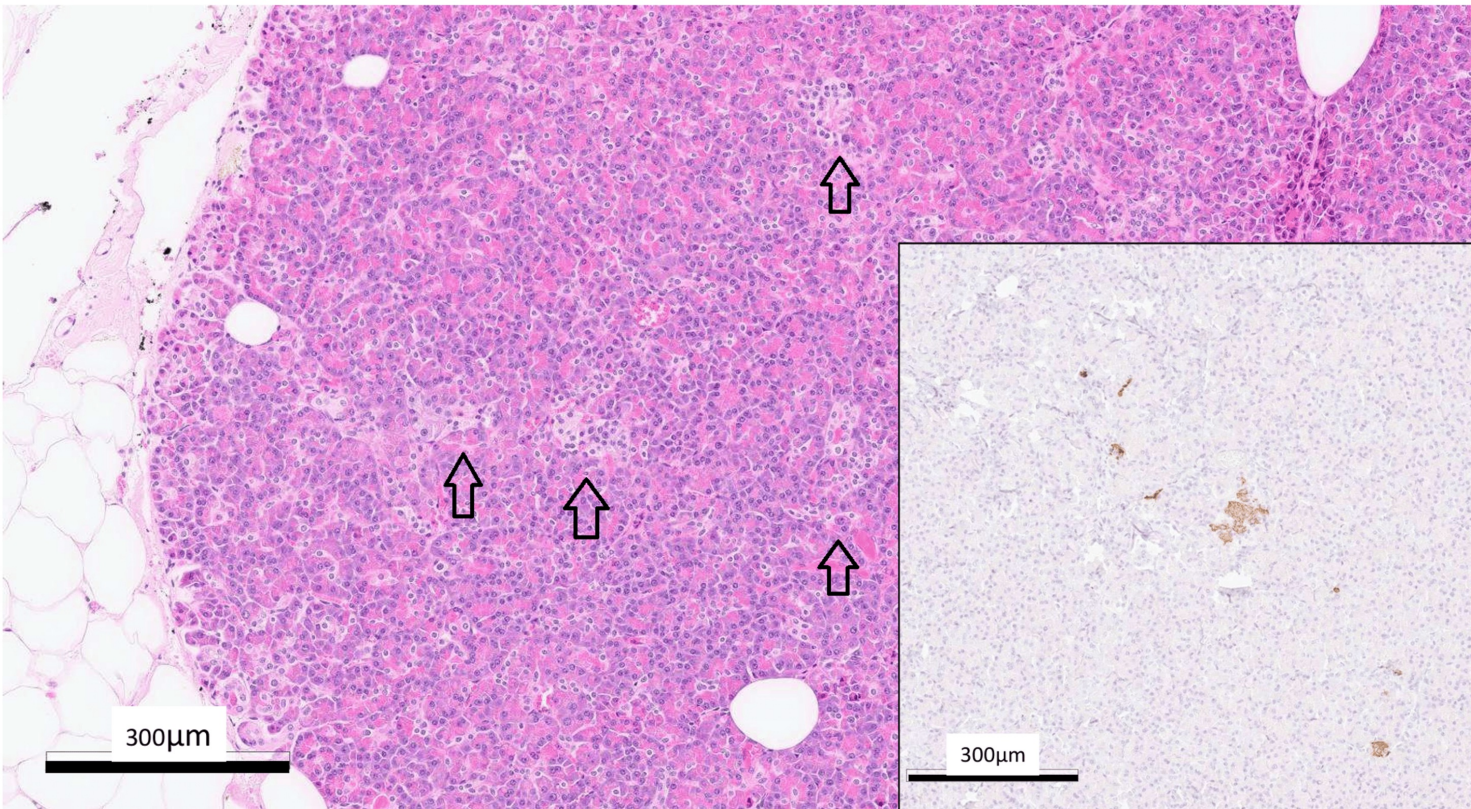
Pancreatic tissue from an individual with a normal distribution of the islet of Langerhans for comparison. Highlighted by insulin immunostaining (inlet). Hematoxylin and eosin staining (x100) and immunoperoxidase (x100). Scale bar =300 μm Arrow: Langerhans Islands. Observe the typical distribution and size of the Langerhans islets in healthy pancreatic tissue.

The observed islet hyperplasia represents a substantial deviation from normal pancreatic architecture (Figure 3). Findings were suitable for the diagnosis of nesidioblastosis.

Postoperatively, a marked improvement in glycemic control was observed, with the patient's daily glucose requirement decreasing significantly to approximately 400 grams. Despite this reduction, occasional dextrose infusions via the PICC line were still necessary to maintain blood glucose levels between 55 and 60 mg/dL. Given the improved glycemic control, the patient was discharged home with a continuous glucose monitoring system (Dexcom) and a protocol for home dextrose infusion as needed. This arrangement was made under close supervision by the treating endocrinologist.

Continuous glucose monitoring data analysis revealed a mean blood glucose of 95 mg/dL, with 94% of readings within the target range. However, severe hypoglycemia was recorded in 3% of the readings, suggesting that completion of total pancreatectomy might be necessary later. Given the extensive nature of the procedure and limited data on total pancreatectomy in pregnant women, the decision was made to postpone this intervention until after delivery.

At 30 weeks of gestation, the patient underwent an elective cesarean section. The neonate was transferred to the neonatal intensive care unit. While the newborn did experience hypoglycemia, it was successfully managed with dextrose infusion during the first few days of life. Importantly, no complications related to hypoglycemia were observed in the neonate.

Postpartum, the pharmacological approach was implemented as an intermediate step before proceeding to the more aggressive total pancreatectomy initially planned. The patient was initiated on a regimen of diazoxide 550 mg daily and intramuscular octreotide 30 mg monthly. Despite adherence to the aforementioned pharmacological treatment, the patient continued to require occasional dextrose infusions. This persistent need for supplementary glucose administration indicates that medication alone was insufficient for fully managing her condition. The multidisciplinary team decided to proceed with the completion of a total pancreatectomy.

The patient was electively admitted for completion of a total pancreatectomy. The procedure was performed without complications. Postoperatively, the patient was closely monitored, and glycemic control was achieved using a continuous subcutaneous insulin infusion pump. She was discharged on postoperative day 10. At the time of this report's publication, excellent glycemic control has been consistently documented through continuous glucose monitoring (Dexcom system). Follow-up assessments at the outpatient clinic on two, four, eight, and 12 weeks have confirmed both maternal and infant well-being, with the child exhibiting normal growth and development.

## Discussion

The diagnosis and management of nesidioblastosis in pregnant women present significant challenges due to the rarity of such cases and the limited literature available. This case was further complicated by the need to differentiate between various hypoglycemic disorders, made more complex by the patient's previous surgical history and few available radiological/endoscopic tests.

The intraoperative findings, which revealed no palpable masses or lesions detectable via intraoperative ultrasound, further strengthened the diagnosis of NIPHS, aligning with recent literature, which indicates that intraoperative inspection and palpation correctly localize insulinomas in 91% of cases. In comparison, intraoperative ultrasound achieves 93% accuracy [5].

The multidisciplinary team considered two primary pharmacological interventions: somatostatin analogs (e.g., octreotide) and diazoxide. Both medications can decrease insulin secretion through different mechanisms. Somatostatin analogs bind to somatostatin receptors, inducing cell membrane hyperpolarization by activating potassium channels [6]. Diazoxide activates ATP-sensitive potassium channels in pancreatic beta cells, leading to membrane hyperpolarization [7]. This hyperpolarization reduces calcium influx in both instances, inhibiting insulin-containing vesicles' exocytosis.

Several case reports have discussed the efficacy of octreotide in managing insulin secretion during pregnancy. Boulanger et al. reported a case of a 36-year-old pregnant woman with nesidioblastosis treated successfully with octreotide infusions. The initial dose of 1000 μg/day was gradually reduced during the last trimester, resulting in adequate glycemic control and a normal birth at 32 weeks via elective cesarean section [8]. The newborn exhibited no malformations and demonstrated normal postnatal development. However, contrasting outcomes have been reported. Geilswijk et al. presented a case of a 34-year-old pregnant woman treated with octreotide, where the fetus experienced growth retardation [9]. Furthermore, given octreotide's known placental transfer, some case reports have suggested a potential link between its use and an increased risk of necrotizing enterocolitis in neonates [10]. Concerning the teratogenic effects of diazoxide, there is a scarcity of data specifically addressing its impact on pregnant women. However, animal experiments have revealed potential teratogenic risks. In these studies, diazoxide was associated with various malformations, including cardiac anomalies and degeneration of pancreatic beta cells [11]. After presenting the pros and cons of the available medical therapeutic options, the patient rejected this option outright. Thus, we concede with the surgical approach.

Concerns surrounding pancreatic surgery during pregnancy primarily stem from the procedure's extent and potential risks to both the mother and fetus. A comprehensive systematic review by Fogliati et al., published in 2022, provided valuable insights into this complex issue by examining pancreatic surgery for pancreatic cystic neoplasms in pregnancy. The review analyzed 47 case reports of pregnancy-associated pancreatic cysts; notably, 43% of surgeries were performed after delivery, indicating a preference for postponing intervention when possible. Only 7% of patients underwent total pancreatectomy. The review also reported that 14% of pregnancies resulted in abortion, both voluntary and involuntary, underscoring the potential complications associated with these cases [12].

This case report presents the challenging management of a pregnant woman experiencing hypoglycemia who was eventually diagnosed with nesidioblastosis. The limited literature on the subject significantly impacted the treatment approach, necessitating the involvement of a wide range of specialists to address each aspect of this complex case.

The decision to proceed with an elective cesarean delivery at only 30 weeks of gestation carried significant risks, but the potential adverse effects of recurrent severe maternal hypoglycemia on fetal well-being left few alternatives. Additionally, the planned completion of total pancreatectomy, an extensive and rarely documented procedure in this context, further complicated the decision-making process. Given these circumstances, an ethics committee was consulted and played a crucial role in the decision-making process for this case.

After delivery, therapeutic options became variable and available. Medical management was initiated to fail later to reach optimal glycemic control and eliminate recurrent severe hypoglycemic episodes. Thus, completion of a total pancreatectomy was contemplated.

Reviewing the current English literature demonstrates that this is the first reported case of a pregnant patient who suffered nesidioblastosis and was treated by surgical means of partial pancreatic resection to alleviate hypoglycemia episodes and decrease dextrose dependence.

## Conclusions

This case presented significant complexities in both diagnosis and treatment, which were effectively managed through the collaborative efforts of a multidisciplinary team. The team's comprehensive approach addressed all aspects of this complex medical condition. Crucial to the successful outcome was carefully considering the timing and extent of surgical intervention. Equally important was the attention paid to the patient's preferences and individual autonomy.
